# Evaluation of dentinal tubule penetration of a novel nano calcium hydroxide–zinc oxide intracanal medicament: An in vitro pilot study

**DOI:** 10.1016/j.jobcr.2026.101565

**Published:** 2026-10-29

**Authors:** K. Chowdhury, S.D.P. Antony, A Sanghavi, P.K. Elango, P. Solete

**Affiliations:** a Department of Conservative Dentistry and Endodontics, Saveetha Dental College and Hospitals, Saveetha Institute of Medical and Technical Sciences, Saveetha University, 162, PH Road, Chennai, 600077, Tamil Nadu, India; b Department of Faculty of Dental Medicine and Oral Health Sciences, McGill University, Montreal, Quebec, Canada

**Keywords:** Confocal laser scanning microscope, Dentinal tubule penetration, Intracanal medicaments, Innovation, Nano calcium hydroxide, Triple antibiotic paste, Zinc oxide

## Abstract

**Background:**

Residual microorganisms within dentinal tubules remain a key factor in persistent endodontic infections. Therefore, the ability of intracanal medicaments to effectively penetrate these tubules is critical for post-endodontic treatment success and effective long-term prognosis.

**Aim:**

To evaluate and compare the dentinal tubule penetration capabilities of a novel nano calcium hydroxide–zinc oxide (NCAL + ZnO) formulation with conventional calcium hydroxide (RC CAL) and triple antibiotic paste (TAP) using confocal laser scanning microscopy (CLSM).

**Materials and methods:**

Forty-five extracted single-rooted human teeth were biomechanically prepared using standardized protocols and randomly allocated into three groups (n = 15). Medicaments were labelled with rhodamine B and placed into root canals. Samples were sectioned at coronal, middle, and apical thirds and analyzed using CLSM. Penetration depth (μm) was measured and analyzed using one-way ANOVA and Tukey post hoc test (p < 0.05).

**Results:**

RC CAL demonstrated the greatest penetration depths in the coronal and middle thirds, while NCAL + ZnO was the only material showing penetration in the apical third. All intergroup differences were statistically significant (p < 0.001).

**Conclusion:**

The novel nano Ca(OH)_2_–ZnO formulation showed a distinct advantage in apical penetration, suggesting potential clinical relevance for improving disinfection in anatomically challenging apical regions.

## 1 Introduction

Successful endodontic therapy depends on eliminating microorganisms from the root canal system and preventing reinfection caused by residual pathogenic persistent microorganisms. However, achieving complete disinfection of the root canal system continues to remain a challenge due to complex anatomy including the presence of lateral canals, fins, isthmuses, and dentinal tubules.^1^, ^2^, ^3^ Among these, dentinal tubules serve as critical reservoirs for microbial colonization. These microscopic channels extend from the pulp towards the dentino-enamel junction and provide a suitable environment for bacterial proliferation.^4^ Microorganisms such as *Enterococcus faecalis* and *Actinomycetes* that are strongly associated with persistent endodontic infections, penetrate dentinal tubules up to depths of 500–1000 μm.^5^, ^6^ This penetrating capability contributes to endodontic treatment failure and the development of post-treatment periapical disease^1^^.^ Furthermore, the elimination of bacteria from dentinal tubules is complicated by the buffering capacity of dentin, which reduces the effectiveness of antimicrobial agents by limiting the penetration of irrigants beyond the main canal space.^7^, ^8^ As a result, conventional biomechanical preparation alone is not sufficient to achieve complete disinfection, and the use of intracanal medicaments becomes necessary.^9^

Calcium hydroxide has traditionally been regarded as the gold standard intracanal medicament due to its high alkalinity and broad antimicrobial properties.^10^ Its antimicrobial effect is attributed to the release of hydroxyl ions which disrupts bacterial cell membrane and inactivates essential enzymes.^11^ However, its effectiveness depends on its ability to diffuse into dentinal tubules and maintain adequate concentration over time. This is often influenced by several factors including particle size, solubility, vehicle, and dentinal sclerosis.^12^ Another intracanal medicament that is widely used in endodontics is the Triple antibiotic paste (TAP). This intracanal medicament is a combination of ciprofloxacin, metronidazole, and minocycline and has a potent antimicrobial action against polymicrobial infections.^13^ Nevertheless, concerns remain regarding its depth of penetration, viscosity, and potential adverse effects such as dentin discoloration and cytotoxicity.^14^

With advances in nanotechnology, the development of nanoparticle-based intracanal medicaments and sealers has opened new possibilities in endodontic therapy. Nanoparticles possess unique physicochemical properties, including increased surface area and enhanced activity, which may improve the ability to penetrate dentinal tubules and interact with microbial biofilms.^15^ Zinc oxide nanoparticles have demonstrated intrinsic antimicrobial activity and favourable flow properties, making them a promising addition to calcium hydroxide-based formulations.^16^ When combined, these materials may improve dentinal penetration and exhibit synergistic antimicrobial activity. This may be useful in challenging regions such as the apical third of the root canal, where dentinal tubules are fewer in number and have a smaller diameter.^17^ Confocal laser scanning microscopy (CLSM) is a reliable technique for evaluating dentinal tubule penetration, as it enables high-resolution, three-dimensional visualization of fluorescently labelled materials within dentin while preserving specimen integrity.^18^

Despite the growing interest in nanoparticle-based intracanal medicaments, there is limited evidence regarding their penetration ability in comparison with conventional agents, especially in the apical third of the root canal. Therefore, the present study aimed to evaluate and compare the dentinal tubule penetration of a novel nano calcium hydroxide–zinc oxide formulation with conventional calcium hydroxide and triple antibiotic paste using CLSM.

## 2 Materials and Methods

### 2.1 Study design

This in vitro pilot study was designed to evaluate the dentinal tubule penetration of the novel nano Ca(OH)_2_–ZnO formulation in comparison with traditionally used intracanal medicaments. Ethical approval was obtained from the Institutional Review Board with the reference number **SRB/SDC/ENDO-2305/24/TH-77** prior to the commencement of the study.

### 2.2 Sample selection and preparation

As this investigation was designed as a pilot in vitro study, a formal sample size calculation was not performed. A sample size of 15 specimens per group was selected based on previous CLSM dentinal tubule penetration studies reported in the endodontic literature.Teeth with cracks, root caries, internal or external resorption, calcifications, or previous endodontic treatment were excluded. All soft tissue remnants and calculus deposits were removed using ultrasonic scalers. The specimens were stored in 0.1% thymol solution to prevent dehydration and microbial contamination until further use. The crowns were sectioned using a diamond disc under continuous water cooling to standardize the root length (Fig. 1). Working length was determined by inserting a #10 K-file into the canal until it was just visible at the apical foramen and subtracting 1 mm from this measurement.

**Fig. 1:**
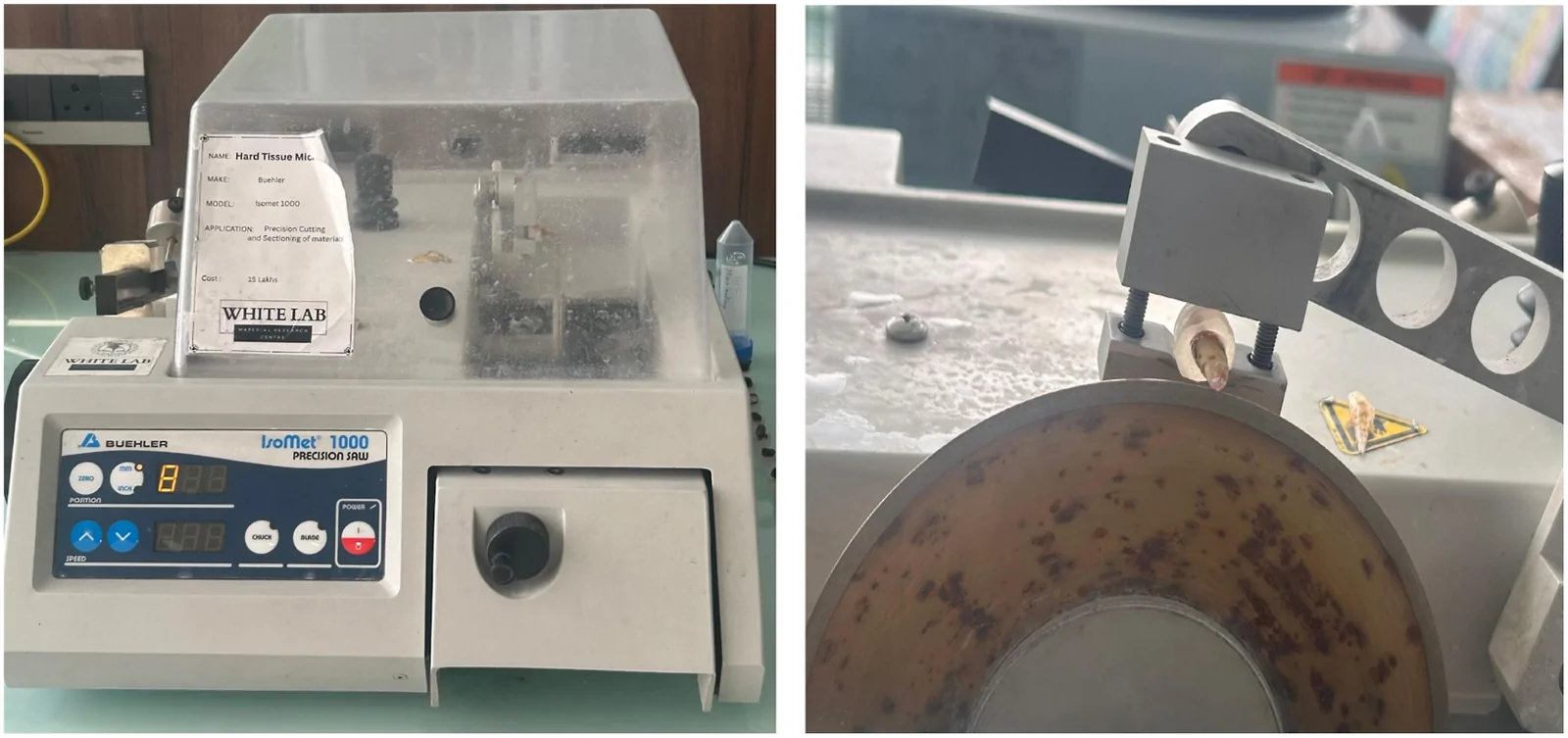
Tooth specimen mounted and sectioned using a diamond disc in the IsoMet™ 1000 Precision Saw for dentinal tubule penetration evaluation.

### 2.3 Biomechanical preparation

Root canal instrumentation was carried out using rotary nickel–titanium files (ProTaper Universal system) up to size F3. During instrumentation, canals were irrigated with 3% sodium hypochlorite using a side-vented needle. Following instrumentation, a final irrigation protocol was carried out using 17% EDTA for 1 min to remove the smear layer, followed by a final rinse with distilled water to eliminate the residual chemicals. The canals were then dried using sterile paper points.

### 2.4 Grouping of samples

The prepared samples were randomly allocated into three experimental groups (n = 15 per group). Group I consisted of nano calcium hydroxide–zinc oxide (NCAL + ZnO).Group II consisted of commercial calcium hydroxide paste (RC Cal; Prime Dental Products Pvt Ltd, India). Group III consisted of triple antibiotic paste (ciprofloxacin, metronidazole, and minocycline mixed in a 1:1:1 ratio).

### 2.5 Preparation and placement of medicaments

The novel nano calcium hydroxide–zinc oxide (NCAL + ZnO) formulation was prepared using nano-sized calcium hydroxide and zinc oxide particles synthesized by the sol–gel method, as previously described and characterized by Chowdhury et al..^17^ Briefly, nano-sized Ca(OH)_2_ particles were synthesized from calcium salts dissolved in isopropyl alcohol, followed by gel formation, drying, and calcination. Calcium hydroxide precipitation was performed under alkaline conditions (pH 12–13) at 60–70°C. Nano-sized ZnO particles were similarly synthesized using the sol–gel method, followed by calcination at 300–400°C. The resulting nano-Ca(OH)_2_ and ZnO powders were combined in a 1:1 wt ratio and homogenized using a mechanical stirrer to obtain a uniform formulation. Dynamic light scattering analysis demonstrated a particle size range of 45–55 nm, while zeta potential analysis revealed a surface charge of 20.39 mV. Detailed physicochemical characterization of the formulation has been previously reported.

Each intracanal medicament was mixed with 0.1% (w/v) rhodamine B dye to facilitate fluorescent visualization of medicament penetration under CLSM.^19^ The concentration was selected to provide adequate fluorescence while minimizing potential alteration of the physicochemical properties of the tested medicaments.The medicaments were introduced into the root canals using a lentulospiral at low speed to ensure uniform distribution along the canal walls, while minimizing void formation. The canal orifices were sealed with a temporary restorative material to prevent contamination and leakage. All specimens were stored at 37°C and 100% humidity for 7 days to simulate intraoral conditions and allow adequate penetration of the medicaments into dentinal tubules.

### 2.6 Sectioning of samples

Following the incubation period, each specimen was sectioned horizontally using a diamond disc under water cooling. Sections were obtained at three standardized levels including the coronal third, middle third and apical third. Each section was approximately 1 mm thick to allow visualization under microscopy.

### 2.7 Confocal laser scanning microscopy (CLSM) analysis

The sections were examined using a confocal laser scanning microscope (Model Stellaris 5 WLL, Manufacturer Leica, Country Switzerland) equipped with appropriate fluorescent filters for rhodamine B visualization. Rhodamine B was excited using a 561 nm laser, and fluorescence emission was collected between 570 and 620 nm. Digital images were captured at a magnification of 10× and analyzed using Las X software. Dentinal tubule penetration depth was measured in micrometers (μm). Penetration depth was measured at three approximately equidistant circumferential locations around each root section. The maximum penetration depth recorded among the measurements was considered the representative penetration depth for each specimen and was used for statistical analysis. Subsequently, the specimen-level values were averaged to calculate the mean penetration depth and standard deviation for each experimental group. For the purpose of analysis, a penetration depth of 0 μm was recorded when no rhodamine B fluorescence attributable to medicament penetration beyond the canal wall was detectable within the examined dentinal region under the predefined CLSM imaging conditions. Such measurements were considered non-detectable rather than evidence of complete absence of medicament at the microscopic level.

### 2.8 Statistical analysis

The collected data were tabulated and analyzed using SPSS software. One-way analysis of variance (ANOVA) was used to compare mean penetration depths among the three groups, followed by Tukey's post hoc test for pairwise comparisons. A p-value of < 0.05 was considered statistically significant.

## 3 Results

### 3.1 Dentinal tubule penetration analysis

The mean depth of dentinal tubule penetration for all experimental groups at the coronal, middle, and apical thirds is presented in Table 1.

#### 3.1.1 Coronal third

In the coronal third, RC CAL demonstrated the highest mean penetration depth (971.08 ± 8.01 μm), followed by TAP (533.36 ± 6.96 μm), while NCAL + ZnO showed the least penetration (358.12 ± 5.77 μm). The difference among the groups was statistically highly significant (p < 0.001).

#### 3.1.2 Middle third

A similar trend was observed in the middle third. RC CAL exhibited the greatest penetration (965.68 ± 10.09 μm), followed by TAP (529.24 ± 7.72 μm), and NCAL + ZnO (355.96 ± 6.04 μm). The intergroup differences were statistically highly significant (p < 0.001).

#### 3.1.3 Apical third

In contrast, the apical third showed a distinct pattern. Penetration was observed only in the NCAL + ZnO group (351.08 ± 6.03 μm), whereas both RC CAL and TAP demonstrated no measurable penetration (0.00 ± 0.00 μm). This difference was statistically highly significant (p < 0.001) (see Table 2).

RC CAL demonstrated the greatest penetration depths in the coronal and middle thirds, while NCAL + ZnO was the only material showing measurable penetration in the apical third. At the apical level, NCAL + ZnO differed significantly from both RC CAL and TAP, whereas RC CAL and TAP did not differ significantly.

### 3.2 Statistical comparison

**Table 1:** Mean ± SD and intergroup comparison.

Section	Group	Mean ± SD (μm)	p-value
Coronal	RC CAL	971.08 ± 8.01	<0.001
	NCAL + ZnO	358.12 ± 5.77	
	TAP	533.36 ± 6.96	
Middle	RC CAL	965.68 ± 10.09	<0.001
	NCAL + ZnO	355.96 ± 6.04	
	TAP	529.24 ± 7.72	
Apical	RC CAL	0.00 ± 0.00	<0.001
	NCAL + ZnO	351.08 ± 6.03	
	TAP	0.00 ± 0.00	

The results indicate statistically significant differences in penetration depth among all groups at each level of the root canal (p < 0.001).

### 3.3 Post hoc analysis

**Table 2:** Pairwise comparison of penetration depth (Tukey test).

Section	Comparison	Mean Difference	95% CI	p-value
Coronal	RC CAL vs NCAL + ZnO	612.96	608.28 – 617.64	<0.001
	RC CAL vs TAP	437.72	432.55 – 442.89	<0.001
	NCAL + ZnOvs TAP	−175.24	−179.60 to −170.88	<0.001
Middle	RC CAL vs NCAL + ZnO	609.72	603.91 – 615.53	<0.001
	RC CAL vs TAP	436.44	430.35 – 442.53	<0.001
	NCAL + ZnO vs TAP	−173.28	−178.07 to −168.49	<0.001
Apical	RC CAL vs NCAL + ZnO	−351.08	−353.57 to −348.59	<0.001
	NCAL + ZnOvs TAP	351.08	348.59 – 353.57	<0.001
	RC CAL vs TAP	0.00	0.00 - 0.00	1.000

At the apical level, statistically significant differences were observed between NCAL + ZnO and RC CAL and between NCAL + ZnO and TAP (both p < 0.001), whereas no statistically significant difference was observed between RC CAL and TAP (p = 1.000).

### 3.4 Qualitative CLSM observations

CLSM images demonstrated distinct penetration patterns among the tested medicaments. RC CAL showed dense and deep penetration in the coronal and middle thirds. TAP exhibited moderate penetration in these regions. NCAL + ZnO showed consistent penetration in the apical third, with visible intratubular diffusion (Fig. 2 and Fig. 3a, Fig. 3b, Fig. 3ca, 3b and 3c).

**Fig. 2:**
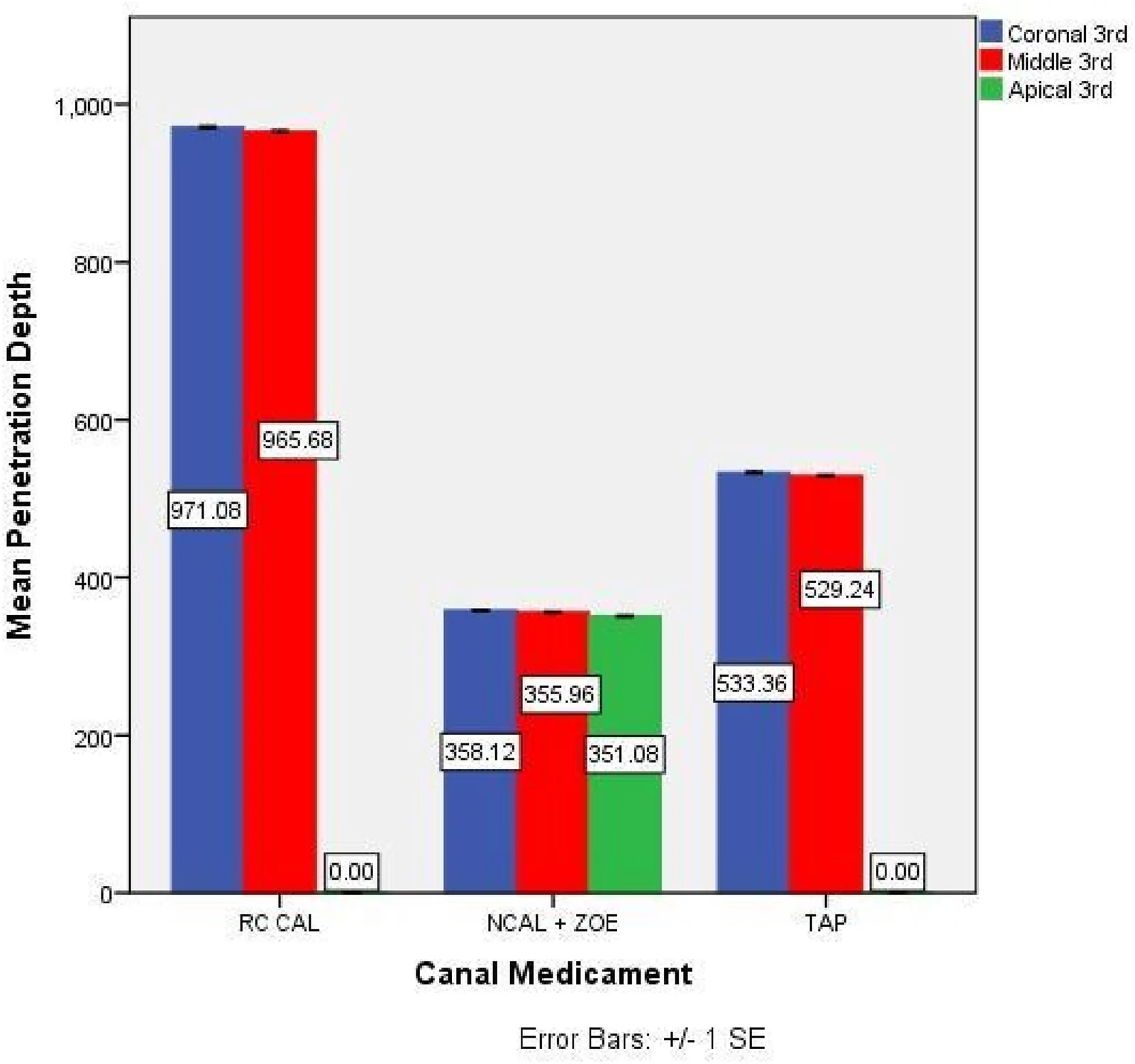
Graph showing the comparative penetration depth (μm) among the study groups, demonstrating variations in the ability of the tested materials to penetrate dentinal tubules. Higher values indicate greater depth of penetration.

**Fig. 3a:**
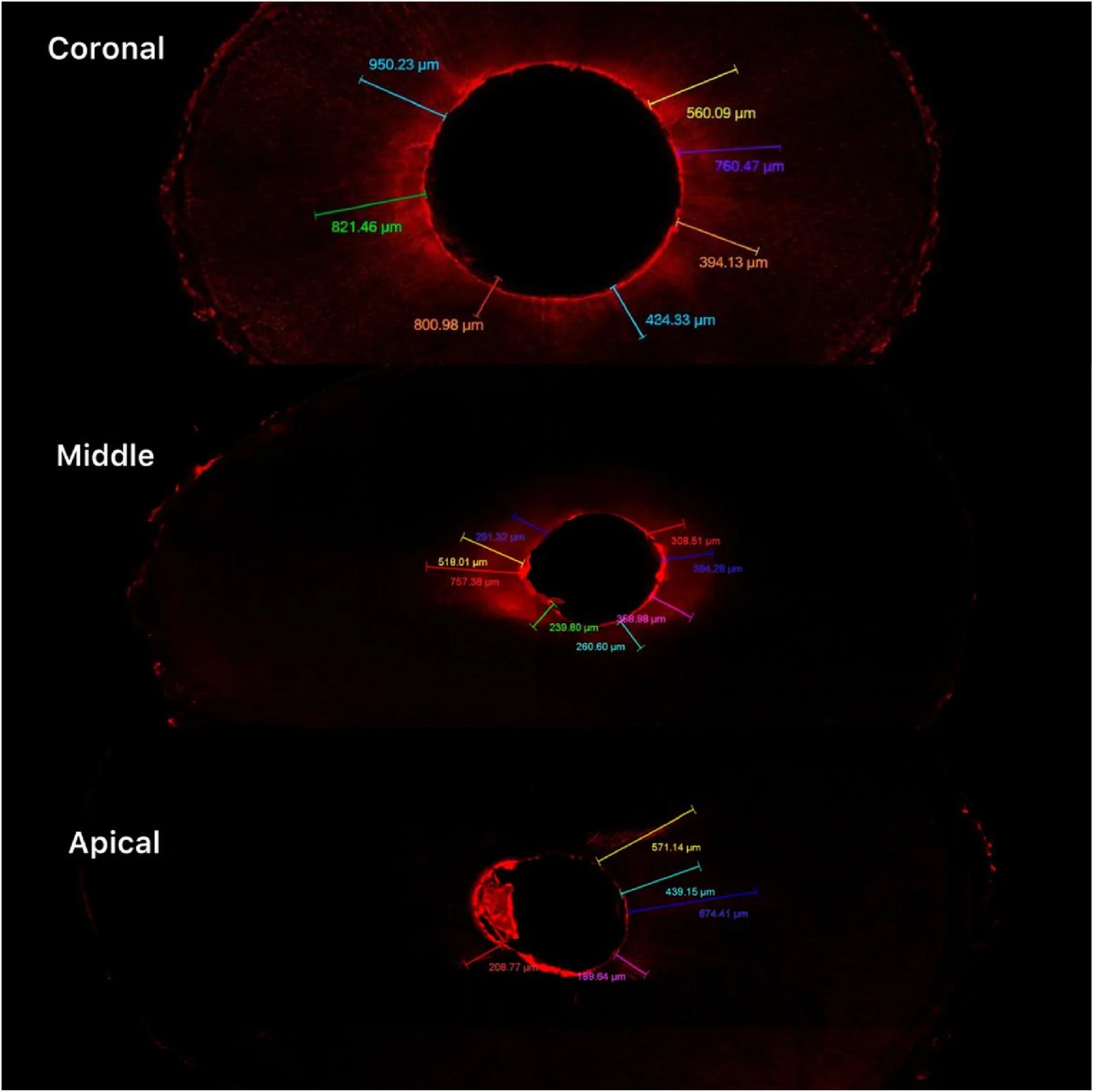
Representative confocal laser scanning microscopy (CLSM) images of the nano calcium hydroxide–zinc oxide (NCAL + ZnO) intracanal medicament demonstrating dentinal tubule penetration at the coronal, middle, and apical thirds of the root canal.

**Fig. 3b:**
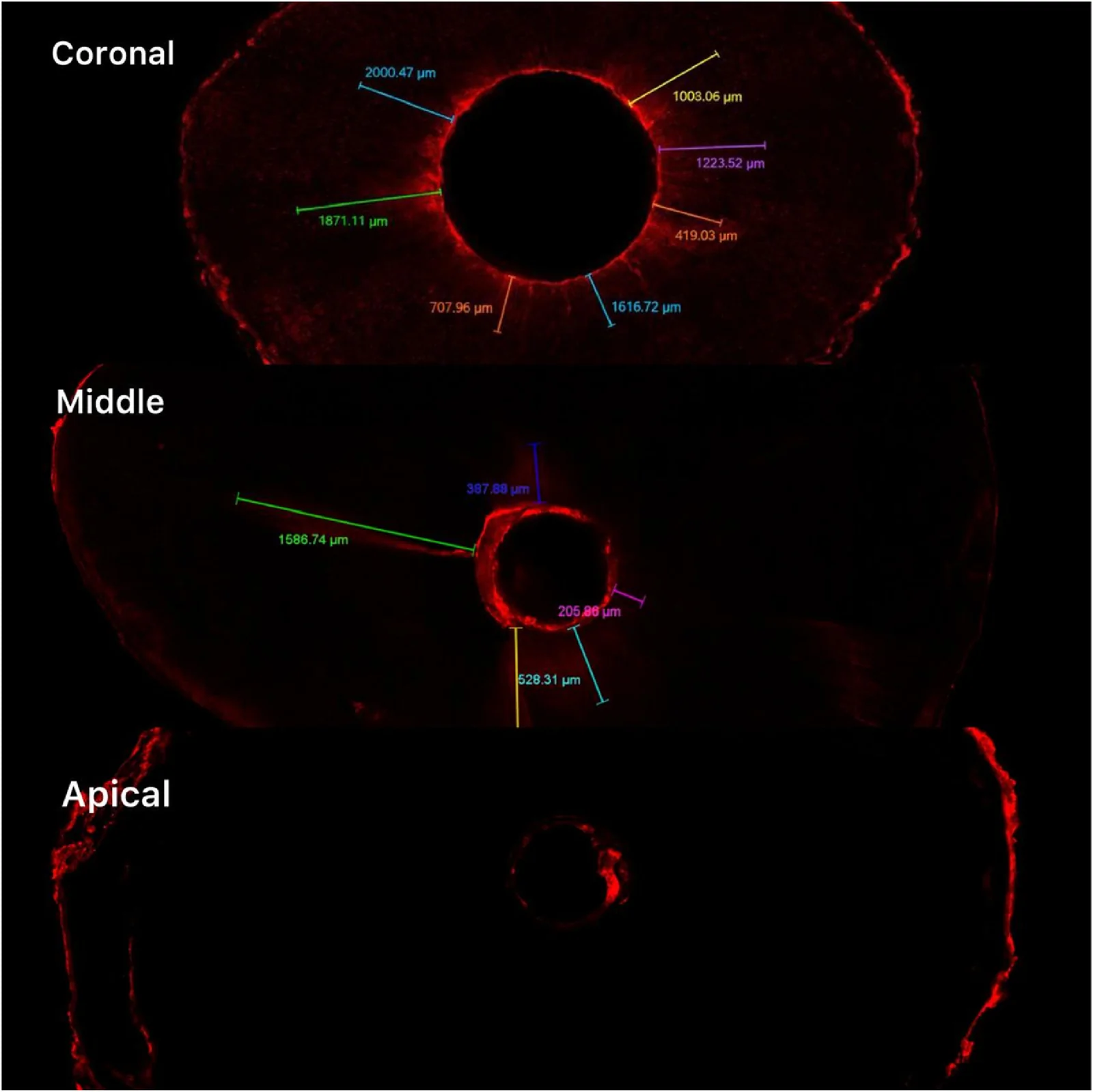
Representative confocal laser scanning microscopy (CLSM) images of the commercial calcium hydroxide medicament (RC CAL) demonstrating dentinal tubule penetration only at the coronal and middle third of the root canal.

**Fig. 3c:**
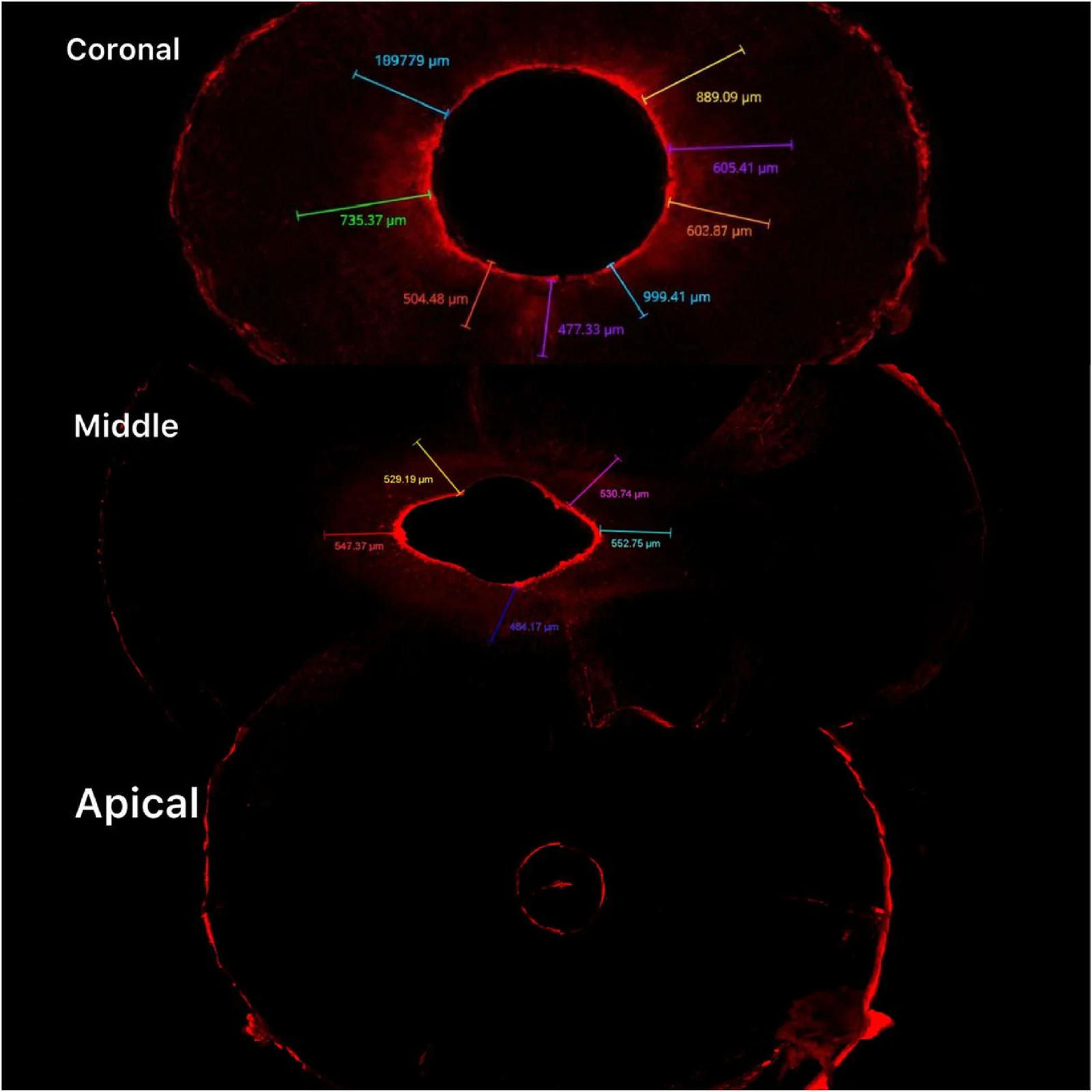
Representative confocal laser scanning microscopy (CLSM) images of the Triple Antibiotic Paste (TAP) group demonstrating dentinal tubule penetration only at the coronal and middle of the root canal.

## 4 Discussion

Dentinal tubule penetration is a critical factor influencing the antimicrobial efficacy of intracanal medicaments. Microorganisms residing within dentinal tubules are often protected from mechanical instrumentation, chemical agents and irrigants, making deep penetration necessary for effective disinfection.^19^, ^20^ CLSM is a validated technique for evaluating such penetration due to its ability to visualize fluorescently labelled materials within dentin in a non-destructive manner.^21^ In the present study, clear differences in the penetration depth were observed among the tested medicaments at all levels of the root canal. Notably, the nano Ca(OH)_2_–ZnO formulation was the only material that demonstrated penetration into the apical third.

Commercial calcium hydroxide (RC CAL) demonstrated the highest penetration in the coronal and middle thirds, consistent with the findings reported in previous studies. This can be explained by its ability to dissociate into calcium and hydroxyl ions, which facilitates diffusion through dentinal tubules.^10^, ^11^, ^22^ In addition, the relatively larger diameter and higher density of dentinal tubules in the coronal and middle thirds likely contribute to the penetration of the medicament in these regions.^23^ Triple antibiotic paste (TAP) on the other hand demonstrated moderate penetration in the coronal and middle thirds. Although TAP is known for its strong antimicrobial properties, its penetration capacity may be limited due to its viscous consistency and complex molecular composition.^24^ Additionally, interactions between the antibiotic components and dentin may further influence diffusion behaviour, resulting in reduced penetration compared to calcium hydroxide.^13^

A key finding of this study was the ability of the novel nano calcium hydroxide–zinc oxide formulation to penetrate the apical third. The apical third is recognized as the most difficult region to disinfect due to complex anatomy including reduced tubule diameter, lower tubule density, and increased sclerosis.^25^ The improved penetration observed with the nano formulation can be attributed to small particle size, which allows it to flow into narrower dentinal tubules more easily.^15^ Furthermore, zinc oxide nanoparticles contribute to improved flow characteristics and dispersion, facilitating better adaptation to the confined apical anatomy.^16^ The increased surface area-to-volume ratio of nanoparticles also enhances their interaction with dentinal walls, potentially improving penetration depth.^26^

A noteworthy observation in the present study was the regional variation in penetration behaviour. Although the nano Ca(OH)_2_–ZnO formulation exhibited lower penetration depths in the coronal and middle thirds compared with conventional calcium hydroxide, it was the only material that demonstrated measurable penetration in the apical third. The nanoscale particle size of the formulation (45–55 nm) may have facilitated penetration into the narrower dentinal tubules present in the apical region. However, the precise mechanism underlying this regional variation remains unclear and cannot be definitively explained by the present study. Factors such as nanoparticle aggregation, viscosity changes, and interactions with dentinal substrates have influenced penetration behaviour and should be investigated further. Similar findings have been reported in studies evaluating nanoparticle penetration, where enhanced performance was not uniformly observed across all regions of the root canal.^15^, ^27^ Similar findings have been reported in studies evaluating nanoparticle penetration, where enhanced performance was not uniformly observed across all regions of the root canal. The results of the present study also support the well-established trend of decreasing penetration from coronal to apical regions for conventional medicaments.^19^, ^21^ However, the ability of the nano formulation to overcome this limitation and achieve measurable penetration in the apical third represents a clear advantage.

From a clinical perspective, these findings suggest that while conventional calcium hydroxide remains highly effective in the coronal and middle thirds, it may be insufficient in addressing infections in the apical region. The novel nano Ca(OH)_2_–ZnO formulation demonstrated measurable penetration within the apical third, suggesting its potential to improve delivery of intracanal medicaments into anatomically challenging regions. However, the present study evaluated dentinal tubule penetration rather than antimicrobial efficacy. Therefore, any potential improvement in disinfection remains speculative and should be confirmed through dedicated microbiological investigations.This is particularly important in cases of persistent apical periodontitis, where residual microorganisms within dentinal tubules are responsible for treatment failure.^28^ The use of nanoparticle-based intracanal medicaments aligns with the broader shift towards more precise and biologically driven endodontic therapy.^29^, ^30^

The methodological strengths of this study include standardized root canal preparation, irrigation, and smear-layer removal across all specimens, evaluation at the coronal, middle, and apical thirds, and the use of CLSM to assess fluorescently labelled medicament penetration. The novelty of the present study lies in evaluating the nano calcium hydroxide–zinc oxide formulation for dentinal tubule penetration. To the best of our knowledge, this is among the first studies to evaluate the dentinal tubule penetration of a novel nano calcium hydroxide–zinc oxide intracanal medicament using confocal laser scanning microscopy. CLSM was employed as a non-destructive and reliable technique for evaluating dentinal tubule penetration, allowing high-resolution visualization of fluorescently labelled medicaments within dentin. In addition, root canal preparation, irrigation, and smear layer removal were standardized across all specimens to minimize procedural variability. Furthermore, penetration was assessed at the coronal, middle, and apical thirds of the root canal, enabling a comprehensive regional evaluation of medicament behavior throughout the canal system. A statistical limitation was observed at the apical third, where the RC CAL and TAP groups demonstrated identical measurements with no within-group variability. This resulted in zero variance, limiting the applicability of conventional parametric post hoc comparisons. Although these findings indicate complete removal of medicament in both groups, the corresponding inferential statistics should be interpreted cautiously. The absence of measurable penetration in the RC CAL and TAP groups at the apical third should not necessarily be interpreted as complete absence of medicament diffusion. Rather, the recorded 0 μm penetration values may partly reflect limitations related to the sectioning plane and the detection threshold of confocal laser scanning microscopy.

Several limitations should be considered when interpreting these findings. As this was a pilot in vitro study, the sample size was based on previous CLSM studies rather than a formal sample size calculation. Second, the measurement protocol used the maximum penetration depth obtained from three circumferential measurements, which may not fully represent the overall spatial distribution of the medicament within dentinal tubules. Third, the absence of measurable penetration in the RC CAL and TAP groups at the apical third resulted in zero within-group variance, limiting the applicability of conventional parametric statistical comparisons. In addition, the in vitro design may not fully replicate clinical conditions, where factors such as fluid dynamics, host response, and complex canal morphology may influence medicament behaviour. Finally, this study evaluated penetration depth only and did not directly assess antimicrobial efficacy. Future studies should therefore investigate the relationship between penetration and antibacterial efficacy, as well as biocompatibility and clinical performance.

## 5 Conclusion

Within the limitations of this in vitro pilot study, the nano calcium hydroxide–zinc oxide formulation demonstrated measurable penetration into dentinal tubules in the apical third, whereas no measurable penetration was detected for conventional calcium hydroxide or triple antibiotic paste. These findings suggest that the nano formulation may have potential for improved penetration in anatomically challenging regions; however, its antimicrobial efficacy and clinical relevance require further investigation.

## Patient's/guardian's consent

Not applicable.

## Author's contribution statement

Chowdhury K contributed to conceptualisation, data acquisition, laboratory testing and writing of the manuscript. Antony DP supervised the project, contributing to conceptualisation, data acquisition and interpretation, writing of the manuscript, and critical appraisal of the article. Elango PK contributed to conceptualisation, data analysis, interpretation, drafted and critically revised the manuscript. Amee S contributed to conceptualisation, data analysis, interpretation, drafted and critically revised the manuscript. S.Pradeep contributed to data extraction and analysis, interpretation, drafted and critically revised the manuscript.

## Data availability statement

Data available from the corresponding author upon reasonable request.

## Ethical clearance

SRB/SDC/ENDO-2206/24/474.

## Ethical

The present study is an in vitro experimental investigation. No human participants, clinical samples requiring consent, or animal subjects were involved. Hence, ethical committee approval was not required as per the guidelines of the institutional review board and international standards for biomedical.

## Financial support and sponsorship

This research did not receive any specific grant from funding agencies in the public, commercial, or not - for - profit sectors.

## Declaration of competing interest

The authors declare that they have no known competing financial interests or personal relationships that could have appeared to influence the work reported in this paper.
